# A Mathematical Model of the Immune and Neuroendocrine Systems Mutual Regulation under the Technogenic Chemical Factors Impact

**DOI:** 10.1155/2014/492489

**Published:** 2014-04-27

**Authors:** N. V. Zaitseva, D. A. Kiryanov, D. V. Lanin, V. M. Chigvintsev

**Affiliations:** Federal Budget Scientific Institution, Federal Scientific Center for Medical and Preventive Health Risk Management Technologies, Perm 614000, Russia

## Abstract

The concept of the triad regulatory metasystem, which includes the neuroendocrine and immune regulation systems, is currently generally accepted. Changes occurring in each of the regulatory systems in response to the impact of technogenic chemical factors are also well known. This paper presents mathematical models of the immune and neuroendocrine system functioning, using the interaction between these systems in response to bacterial invasion as an example, and changes in their performance under exposure to chemical factors, taking into account the stage of functional disorders in a producing organ, using the performance of the bone marrow as an example.

## 1. Introduction

Regulation processes in the human body act as one of the most important functions aimed at maintaining the equilibrium in the performance of organs, tissues, and cells. The concept of the triad regulatory metasystem [1], which includes the neuroendocrine and immune regulation systems, is currently generally accepted. Furthermore, different regulation systems (endocrine and immune) have mutual regulatory influences and, to a certain extent, are “subordinate” to one another [2–5]. The performance of certain systems (immune, neural, and endocrine) and the “neuroendocrine-immune” regulation in general can be interfered by technogenic chemical factors [6–9]. Since the beginning of immunology as a science, one of the major functions of the immune system has been considered to be the fight against alien genetic materials, including microorganisms, in particular bacteria. Exposure to technogenic chemical factors induces changes in the interaction between the microorganism (a bacterium) and the macroorganism (a human). The effects of chemical factors can be manifested through changes in bacterial pathogenicity as a result of mutagenesis, killing, or, in contrast, through the stimulation of bacterial reproduction and growth [10]. This issue, which can be referred to as direct effects of chemical factors on microorganisms, is not considered in this study. The second issue concerning “indirect effects” is associated with chemical modulation of the host organism functions, primarily the effects of chemical factors on the immune system [10]. This paper is devoted to the issue of indirect effects. There is well-documented epidemiological evidence on increased incidence and severity of infectious diseases in various population groups exposed to technogenic chemical factors [11, 12]. Epidemiological studies are supported by experimental data reported by authors who associate decreased body resistance to infectious agents with the impact of chemical compounds on immune mechanisms [13–15]. It is, however, necessary to mention that, as is indicated above, the immune system has a complex relationship with the neuroendocrine system and thus the indirect effects associated with the interaction between the host and the microorganism may be mediated not only by changes in the immune system but also through the modulation of other regulatory mechanisms that affect the body's immune defense.

It is well known that, starting from a certain age, the human body starts experiencing natural aging processes which occur in different organs and tissues at a different rate. However, the body generally accumulates various pathophysiological disorders and deviations from normal physiological values, which have a deteriorating impact on the effectiveness of body functioning on the whole and, in particular, on the body systems under consideration [11, 16–18]. Exposure to technogenic chemical factors accelerates the accumulation of these disorders and a reduction in the effectiveness of regulatory organs and systems functioning [11, 16, 17]. These processes, which can be designated as “the evolution of functional disorders of organs and systems,” should be taken into account when studying the impact of chemical factors on the interaction between the immune and neuroendocrine systems.

Biology and medicine have developed a traditional approach to the assessment of human health effects from various exposures, in particular, regulatory systems effects. This approach is based on mathematical statistics methods. Despite the fact that these methods allow an effective solution of specific problems, the investigation of processes taking place in the human body from the point of view of systems analysis requires detailed mathematical formulation of the research problem and the use of more sophisticated mathematical models.

One of the current directions in modeling human body processes is the development of forecast mathematical models describing trends in long-term systemic changes taking into account the impact of environmental factors [17].

Modeling of neuroendocrine and immune system processes is made difficult by the complexity, branching, and lack of knowledge of various regulatory mechanisms, which, obviously, leads to problems concerning conceptual and mathematical formulation as well as the identification and verification of parameters. The majority of studies in this field are devoted to biological and mathematical modeling of individual elements of regulatory mechanisms [19–21] which considerably aids understanding of the studied phenomena, although it does not provide a full and systemic knowledge of the internal relationships and the occurring processes.

The purpose of this study is to provide a mathematical description of the regulatory mechanism which is based on the interaction between elements of the neuroendocrine and immune systems in response to bacterial exposure, taking into account the evolution of functional disorders under negative exposure to chemical agents entering the body from the environment.

## 2. Biological Background

**Figure 1:**
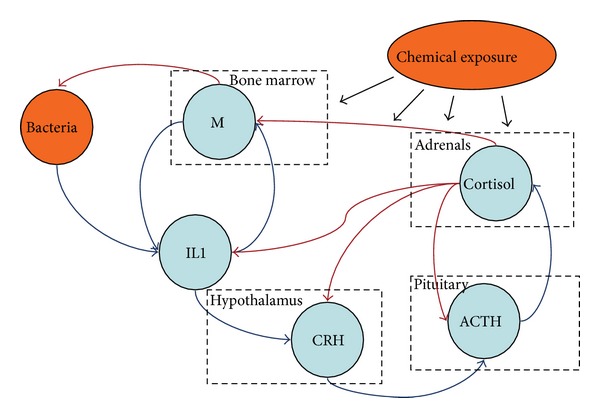
Interaction between the elements of the neuroendocrine and immune systems in response to bacterial invasion (infection). (M-monocytes/macrophages, CRH-corticotropin releasing hormone, and ACTH-adrenocorticotropic hormone. The dashed line rectangles represent producing organs).

The structural diagram of the model, given in Figure 1, consists of several interconnected elements of the neuroendocrine and immune systems involved in the response to bacterial invasion. Each of these elements can be adversely affected by chemical compounds entering the human body from the environment which disrupts the elements' functions leading to regulation failure and effectiveness reduction.

The mechanism of response to bacterial invasion (bacterial infection) is based on the capacity of monocytes and, to a greater extent, of their more mature forms, macrophages, to phagocytosis (attachment, ingestion, and killing) of alien material, including pathogenic bacteria. Since measurement of macrophage count is complicated, in this study, their count is measured by the blood levels of monocytes produced by the bone marrow. The ingestion of infectious agents by monocytes/macrophages (M) is accompanied by synthesis and release of a number of cytokines among which is the proinflammatory interleukin-1 (IL-1) [19]. Elevated IL-1 levels in blood, in their turn, induce numerous regulatory effects as well as the mobilization of monocytes into the infection focus and, by means of specific hypothalamus receptors, stimulate the production of corticotropin releasing hormone (CRH), which stimulates the anterior pituitary to secrete adrenocorticotropic hormone (ACTH). Entering the bloodstream, ACTH stimulates the adrenal glands to produce cortisol. The increase in cortisol levels inhibits ACTH and CRH secretion in a negative feedback loop, stimulates monocyte/macrophage apoptosis, and blocks IL-1 production [20, 21]. In addition, the above mentioned cortisol effects manifest from a particular cortisol level and have a nonlinear character. The negative feedback loop, caused by the increase in cortisol levels maintains the system's equilibrium (homeostasis).

The presented mechanism describes a self-regulating system the performance of which largely depends on the functioning of such organs as the bone marrow, the pituitary gland, the hypothalamus, and the adrenal glands. Functional disorders in the neuroendocrine and immune system organs may lead to regulation failure and an imbalance in their indicators. These disorders can be accumulated over a lifetime both during natural aging of these organs and under the impact of external technogenic chemical factors. Entering the body from the environment, complexes of chemical agents interfere with almost every interaction circuit activating pathological processes [8], including the modification of response to bacterial infection [10].

## 3. Mathematical Description of the Regulation Process

Changes in macrophage count directly depend on monocyte levels in blood; therefore, when the equation is being written, the monocyte/macrophage system is considered. A characteristic of macrophages' existence is programmed death (apoptosis) at elevated cortisol levels [5]. According to the existing knowledge of the mechanism of monocyte production by the bone marrow, monocyte to macrophage maturation and their subsequent death, the equation describing changes in monocyte/macrophage count can be written as follows:

(1) dMdt=β1·(1−D1)+β2·IL-1 ·(1−MMmax⁡)−β3·M·(1+β4·K(t−τ)ncn+K(t−τ)n),

where *M* is monocyte/macrophage count [cells]; *D*_1_ is a parameter of impaired blood-making function of the bone marrow (*D*_1_ ∈ [0; 1]); IL-1 is IL-1 levels in blood [pg/mL]; *M*_max⁡_ is the highest possible monocyte/macrophage count [cells]; *K* is cortisol levels [pg/mL]; *β*_1_ is monocyte/macrophage production rate [cells/min⁡]; *β*_2_ is a parameter characterizing the rate of monocyte migration to the infection focus [cells · mL/(min⁡·pg)]; *β*_3_ is monocyte/macrophage clearance [min^−1^]; *β*_4_ is a parameter characterizing the influence of cortisol on monocyte/macrophage apoptosis; *c* is mean normal cortisol level [pg/mL]; *n* is model nonlinearity parameter; and *τ* is time delay of the effect [min⁡].

The first summand of (1) describes the process of monocyte/macrophage production taking into account a lower rate of their production when the blood-making function of the bone marrow is impaired. The second summand pertains to the effect of the migration of additional monocytes to the infection focus, associated with IL-1 activity. The third summand describes the mechanism of macrophage death taking into account the triggering of apoptosis at high cortisol levels.

Changes in bacterial count are induced by three processes, that is, bacterial entry into the body from the external environment, bacterial growth inside the body, and elimination of bacteria by monocytes/macrophages. Such phenomena are described by equations similar to Lotka-Volterra predator-prey equations [22]:

(2) $$\begin{array}{c} \frac{dP}{dt}=\alpha_{1}+\alpha_{2}\cdot P\cdot \left(1-\frac{P}{P_{\max}}\right)-\alpha_{3}\cdot M\cdot \left(\frac{\alpha_{4}\cdot P}{1+\alpha_{4}\cdot P}\right), \end{array}$$

where *P* is pathogenic bacterial count [CFU/mL]; *P*_max⁡_ is the highest possible bacteria levels [CFU/mL]; *α*_1_ is a parameter of bacterial entry into the body [CFU/mL · min⁡]; *α*_2_ is a parameter of bacterial growth [min^−1^]; *α*_3_ is a parameter of the ingestion of bacteria by monocytes/macrophages [CFU/mL · cells · min⁡]; and *α*_4_ is a parameter characterizing the probability of contact between bacteria and monocytes/macrophages [mL/CFU].

Equation (2) shows that the higher bacterial and macrophage count in the body, the more frequently they interact resulting in bacterial count reduction.

The contact between monocytes/macrophages and bacteria results in IL-1 production which, being an inflammation marker, increases cortisol production levels. Elevated cortisol levels, in their turn, inhibit IL-1 synthesis. An equation of the rates of changes in IL-1 levels in blood is as follows:

(3) dIL-1dt=γ1·M·(α4·P1+α4·P) ·(1−γ2·K(t−τ)ncn+K(t−τ)n)−γ3·IL-1,

where IL-1 is IL-1 levels [pg/mL]; *γ*_1_ is a parameter characterizing IL-1 production rates [pg/mL · cells · min⁡]; *γ*_2_ is a parameter of cortisol's influence on IL-1 production; and *γ*_3_ is IL-1 clearance [min^−1^].

The first summand of (3) characterizes IL-1 synthesis rates during the interaction between monocytes/macrophages and bacteria taking into consideration the effect of IL-1 synthesis inhibition at elevated cortisol levels. The second summand pertains to natural IL-1 clearance.

The appearance of IL-1 in blood accelerates the rates of CRH production by the hypothalamus. Furthermore, the rate at which CHR levels change is influenced by five processes, that is, CHR synthesis by means of IL-1 induced stimulation of the hypothalamus, a decrease in the synthetic function of the hypothalamus, CHR synthesis inhibition by high cortisol levels, fluctuations in hypothalamic activity in accordance with ultradian rhythms, and natural CRH clearance. To describe processes of CHR changes, equations, which had been reported in studies on modeling the hypothalamic-pituitary-adrenal axis [20, 21] and had been modified to take into account the effects of impaired synthetic function of the hypothalamus, were utilized:

(4) dCRHdt=λ1·(1−D2)·(1−λ2·K(t−τ)ncn+K(t−τ)n) +λ3·IL-1−λ4·CRH,

where CRH is CRH levels [pg/mL]; *D*_2_ is a parameter of impaired synthetic function of the hypothalamus (*D*_2_ ∈ [0; 1]); *λ*_1_ is a parameter of CRH production [pg/mL · min⁡]; *λ*_2_ is a parameter of cortisol's influence on CRH production; *λ*_3_ is a parameter of IL-1 influence on CRH production [min^−1^]; and *λ*_4_ is CRH clearance [min^−1^].

The first summand of (4) pertains to reduced CRH production as a result of impaired hypothalamus function and high cortisol levels amid natural ultradian rhythms. The second summand describes IL-1 induced stimulation of CRH production. The last summand pertains to natural CRH clearance from the body.

CRH activates ACTH production by the pituitary gland. It is known that, as cortisol levels increase to a certain level, the pituitary gland stops the release of ACTH. In general terms, an equation describing changes in ACTH production rates is written as follows:

(5) dACTHdt=μ1·(1−D3)·(1−μ2·K(t−τ)ncn+K(t−τ)n) ·CRH−μ3·ACTH,

where ACTH is ACTH levels [pg/mL]; *D*_3_ is a parameter of impaired synthetic function of the pituitary gland (*D*_3_ ∈ [0; 1]); *μ*_1_ is a parameter of ACTH production [min^−1^]; *μ*_2_ is a parameter of cortisol's influence on ACTH production; and *μ*_3_ is ACTH clearance [min^−1^].

ACTH stimulates the adrenal glands to release cortisol. Cortisol release rate is defined by the following equation:

(6) $$\begin{array}{c} \frac{dK}{dt}=\nu_{1}\cdot \left(1-D_{4}\right)\cdot \text{ACTH}\left(t-\tau \right)-\nu_{2}\cdot K, \end{array}$$

where *K* is cortisol levels [pg/mL]; *D*_4_ is a parameter of impaired synthetic function of the adrenal glands (*D*_4_ ∈ [0; 1]); *ν*_1_ is a parameter of cortisol production [min^−1^]; and *ν*_2_ is cortisol clearance [min^−1^].

Equation (6) reflects the process of an increase in cortisol production rates as a result of elevated ACTH levels taking into account the influence of impaired synthetic function of the adrenal gland send time delay.

Thus, the regulation mechanism involving the neuroendocrine and immune systems taking into consideration the time delay is presented as follows:

(7) dMdt=β1·(1−D1)+β2·IL-1·(1−MMmax⁡) −β3·M·(1+β4·K(t−τ)ncn+K(t−τ)n),dPdt=α1+α2·P·(1−PPmax⁡)−α3·M·(α4·P1+α4·P),dIL-1dt=γ1·M·(α4·P1+α4·P) ·(1−γ2·K(t−τ)ncn+K(t−τ)n)−γ3·IL-1,dCRHdt=λ1·(1−D2)·(1−λ2·K(t−τ)ncn+K(t−τ)n) +λ3·IL-1−λ4·CRH,dACTHdt=μ1·(1−D3) ·(1−μ2·K(t−τ)ncn+K(t−τ)n)·CRH−μ3·ACTH,dKdt=ν1·(1−D4)·ACTH(t−τ)−ν2·K.

System of (7) with initial conditions *M*(*t*_0_) = *M*_0_, *P*(*t*_0_) = *P*_0_, IL-1(*t*_0_) = IL-1_0_, CRH(*t*_0_) = CRH_0_, ACTH(*t*_0_) = ACTG_0_, and *K*(*t*_0_) = *K*_0_ is a Cauchy problem formulated for a system of first-order ordinary differential equations with retarded argument. Suppose that all the constants in the equation are nonnegative (it follows from their biological meaning) and *D*_*j*_ are continuous and nonnegative functions of time (*D*_*j*_(*t*)); then, for any nonnegative initial conditions (*M*(*t*_0_) ≥ 0, *P*(*t*_0_) ≥ 0, IL-1(*t*_0_) ≥ 0, CRH(*t*_0_) ≥ 0, ACTH(*t*_0_) ≥ 0, *K*(*t*_0_) ≥ 0), the solution of the problem exists and exists uniquely in the entire function domain (*t* ≥ 0). Furthermore, it follows from the theorem about the existence and uniqueness of solution to the Cauchy problem that, under the assumed conditions for *t* ≥ 0, solution will be continuous and nonnegative (*M*(*t*) ≥ 0, *P*(*t*) ≥ 0, IL-1(*t*) ≥ 0, CRH(*t*) ≥ 0, ACTH(*t*) ≥ 0, and *K*(*t*) ≥ 0).

The complexity and nonlinearity of (7) make it difficult to obtain an analytical solution and lead to the necessity to use numerical methods. For a numerical solution of system (7), a difference scheme based on the fourth-order Runge-Kutta method with a fixed step-length was used [23].

## 4. A Model of Evolution of the Synthetic Function (Changes in Cell or Regulatory Molecule Production) of the Immune and Neuroendocrine Systems under Exposure to Technogenic Chemical Factors

The effectiveness of the mechanism of fighting against bacterial invasion along with the ability of monocytes/macrophages to produce cytokines is also determined by the number of cells involved in the immune response, that is, by the capacity of organs (in this case, the bone marrow) to produce new immune system cells instead of the “used” cells (synthetic function). Impairment of these functions leads to a deficiency in essential cytokines and, as a consequence, to the disturbance of the system homeostasis.

Functional activity of the majority of organs, including the production of regulatory molecules (hormones and cytokines) and cells (monocytes) is known to weaken with age [18, 24, 25], which may correlate with the onset of more severe diseases and a longer treatment and after-care period while environmental chemical factors aggravate such effects [10]. It is necessary to mention that, as is indicated above, the immune system permanently interacts with the neuroendocrine system and thus hormone deficiency or excess, caused by impaired synthetic function of the producing organs, may also disturb the immune response.

In order to describe an aging-related decrease in the functional (synthetic) activity of organs (in this case, those organs producing cells and regulatory molecules), a mathematical model of the evolution of functional disorders in the body's organs and systems under exposure to environmental factors is used [17]. According to this model, impaired synthetic function of the organ (index *j*) is characterized by a parameter of damage *D*_*j*_. *D*_*j*_ ∈ [0; 1]. The value *D*_*j*_ = 0 corresponds to normal (ideal) functioning whereas *D*_*j*_ = 1 corresponds to organ's full functioning failure. The evolution of damage is determined by external impacts and internal disorders due to natural causes (aging). The impacts are understood to be substance intakes, divided by the normal levels, affecting the condition of the body's organs and systems.

Accepting the hypothesis that the rates at which the damage changes due to the impacts of various factors add up, the structure of equations describing the evolution of synthetic function impairments is presented as follows:

(8) $$\begin{array}{c} \frac{dD_{j}}{dt}=a_{j}D_{j}+\overset{n}{\underset{i=1}{\sum }}b_{ji}\langle \frac{p_{i}}{p_{ji}^{N}}-1\rangle , \end{array}$$

where *a*_*j*_ is a coefficient characterizing the rate of synthetic function impairment (damage) of the *j*th organ due to natural causes [1/year]; *b*_*ji*_ is a coefficient characterizing the intensity of the impact of the *i*th negative factor on the damage of the *j*th organ [1/year]; *p*_*i*_ is the intake of the *i*th substance by the human body; *p*_*ji*_^*N*^ is standard (maximum permissible) value of the intake of the *i*th substance for the *j*th organ; and 〈*x*〉 are Macaulay brackets: 〈*x*〉 = 0 at *x* < 0 and 〈*x*〉 = *x* at ≥0.

The presented structure of (8) reflects the general view of the evolution of damage and takes into account macrolevel processes, that is, self-destruction (natural aging) and the accumulation of synthetic function impairments due to nonstandard intakes of substances.

## 5. Identification of Model Parameters

For numerical implementation of the mathematical model obtained in the previous section, it is necessary to identify unknown parameters. The values of these parameters can vary considerably when solving various problems. To present a specific example of modeling, data obtained by a study of the primary immune response to pneumococcal infection was utilized.

The identification of mathematical models describing complex biological processes occurring in the human body is one of the most hard-to-solve problems, the solution of which is attended by considerable uncertainty [26]. In addition, the major problem is associated with the inability to organize a directed experiment, within which indicators would vary over a wide range of values, and the existing system of sample observations is characterized by a significant number of confounding factors. Therefore, the model parameters were determined by structural identification method allowing the use of the published data reported by scientific studies analyzing the interaction between individual elements of the neuroendocrine and immune systems. The missing parameters were evaluated using in-house laboratory testing.

Table 1 presents the identified parameters of the mathematical model of the interaction between the neuroendocrine and immune systems in streptococcal pneumonia (which has been chosen as a simulation example), taking into account the evolution of functional disorders in the bone marrow under negative impacts of chemical agents.

If other types of bacteria or other affecting chemical factors are considered, a further identification of a number of parameters is needed.

## 6. Simulation Results

**Figure 2:**
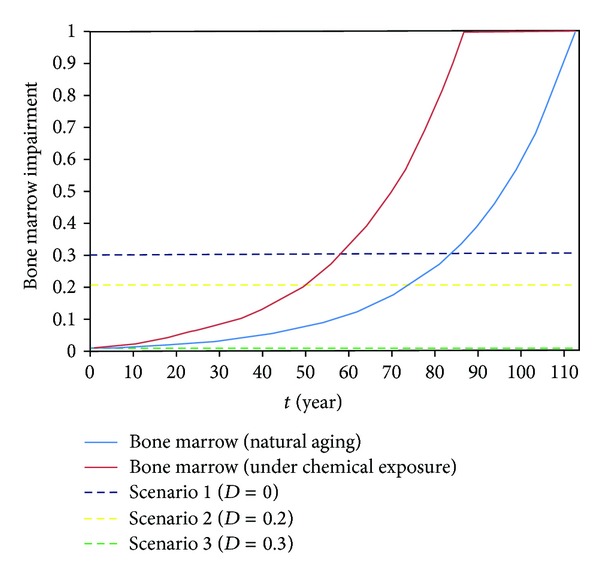
A chart of evolution of impairment of the bone marrow function to produce monocytes due to natural aging and under additional impact of technogenic chemical factors with a specification of scenario levels.

The identified parameters allow us to carry out a numerical experiment showing the behaviour of the obtained model under various scenario conditions which are understood to be various stages of functional disorders in the production of cells or regulatory molecules associated with the impacts of negative chemical factors. The model of impaired synthetic function of the studied organs determines the influence of several factors the major ones of which are chemical substances entering the human body from the environment. One of the possible solutions of (8) is given in Figure 2. The bone marrow has been chosen as an example producing organ as the core element of all the immune responses in the described example is monocytes/macrophages produced by this organ.

This solution reflects the possibility of bone marrow function impairment as a result of natural aging processes and a combined impact of aging and chemical factors. Furthermore, as can be seen from the chart, the impacts of chemical factors can considerably accelerate the accumulation of synthetic function disorders which is supported by our early studies [17].

To check the adequacy of the model, calculations were made for three scenarios varying by the degree of impairment of bone marrow function to produce monocytes associated with the impacts of chemical compounds: *D*_1_ = 0; *D*_1_ = 0.2; and *D*_1_ = 0.3. These values of the damage parameter can be observed at a different age depending on the impacts of negative chemical factors. In addition, the processes of interaction between the elements of the neuroendocrine and immune systems may significantly differ.

**Figure 3:**
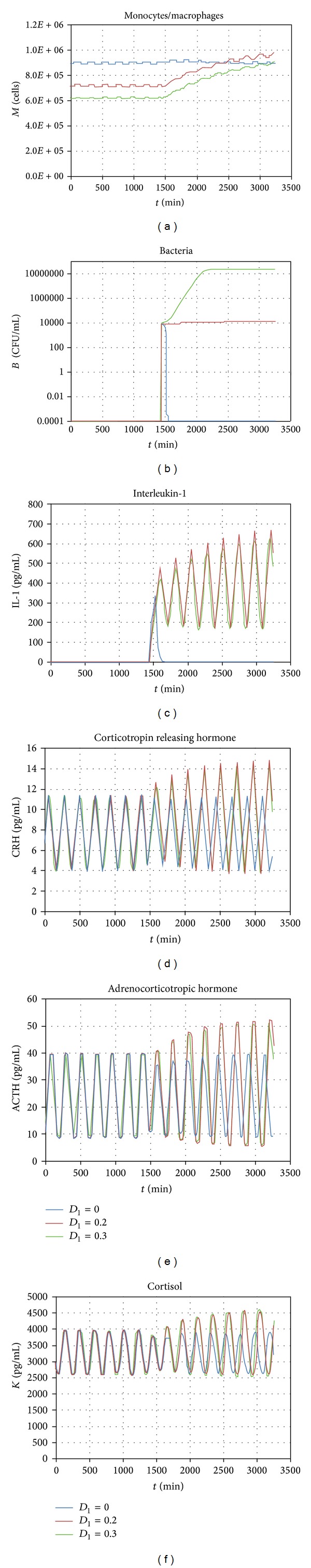
Charts showing changes in (a) macrophage count, (b) bacterial count, (c) IL-1 levels in blood, (d) corticotropin releasing hormone levels in blood, (e) adrenocorticotropic hormone levels in blood, and (f) cortisol levels in blood. The charts show time in hours on the horizontal axis where the curve's starting point corresponds to the point of time of 24 hours before bacterial invasion. The vertical axis shows an indicator's value obtained by a computer simulation. Different colors represent each of the three simulated scenarios with various functional damages to the bone marrow.

Possible solutions of system of (7) are given in Figure 3, which shows changes in three selected variables of the model in case of bacterial infection, that is, monocytes/macrophages, changes in bacterial count, CRH, ACTH, cortisol, and IL-1 levels. The calculation period of the modeling is 2 days. The Appendix contains the scenarios with various functional damages to the hypothalamus, the pituitary, and the adrenal glands.

Each scenario implies the loss of the system's equilibrium, setting the bacterial level of initial streptococcal infection at 10^4^ CFU. This dose obtained using experimental data [5] is capable of inducing the activation of immune system reserves with a demonstrable manifestation of interaction between the neuroendocrine and immune system elements. At a dose of bacteria one order of magnitude less, a healthy body is able to cope with bacterial invasion over a short period of time using local resources and has an insignificant influence on the regulatory system. At a bacterial invasion one order of magnitude more than the selected dose, even a healthy body experiences an exponential bacterial growth which the body cannot cope with and which leads to a total failure of the defense mechanisms.

At the initial stage (1440–1540 minutes), all the scenarios demonstrated an increase in macrophage/monocyte count and triggering of regulatory mechanisms. The difference lies in a reduction in the ability of the bone marrow to produce a sufficient number of monocytes/macrophages (synthetic function) for the second and third scenarios resulting in decreased monocyte/macrophage levels (Figure 3(a)) which, in its turn, reduces the effectiveness of the response to bacterial growth.

The first scenario simulates the system's behavior when the bone marrow synthetic function is not impaired (Figure 2, the green dashed line). After bacterial invasion, the system equilibrium is restored 100 minutes later which corresponds to the elimination of bacterial infection and the normalization of indicators (Figure 3, the blue line). The process under consideration takes place amid a stable ultradian rhythm of changes in the immune and neuroendocrine system indicators. In clinical practice, such changes correspond to either the absence of disease symptoms or acute inflammation resulting in quick recovery (the curing and recovery scenario).

The second scenario simulates a minor impairment of the synthetic function of the bone marrow when the balance between two processes, that is, bacterial growth and their elimination by macrophages, is maintained. The level of synthetic function impairment in this scenario is presented in Figure 2. As can be seen from the chart, the scenario conditions take place much earlier when the bone marrow is influenced by chemical factors. Furthermore, bacterial count does not increase and the immune system continues to be stressed (Figure 3, the red line). In clinical practice, the examples of such conditions can be an exacerbation or remission of a chronic disease (the chronic disease scenario).

The third scenario simulates a significant impairment of monocyte/macrophage production by the bone marrow. The level of the synthetic function impairment is given in Figure 2. This condition takes place at an older age in comparison with the first and second scenarios; however, this level of impairments can be observed at an earlier age in case of a negative impact of chemical factors on the bone marrow. Changes in IL-1 levels differ insignificantly from the second scenario due to an adequate performance of the neuroendocrine system elements. One can observe an unlimited bacterial growth associated with reduced monocyte/macrophage levels due to their reduced production by the bone marrow which can be caused by the impact of environmental chemical factors (Figure 3, the green line). This results in the suppression of all the regulatory indicators manifesting through severe acute conditions and a severe exacerbation of the chronic infection (the severe infection scenario) which may result in death.

## 7. Conclusions and Future Research

This paper considers a mathematical model which allows the description of the mechanism of regulating the neuroendocrine and immune system elements in response to bacterial invasion and takes into account the evolution of functional disorders of the elements under the impact of environmental factors. This work shows the performance of individual structural elements of the systems under consideration, the description of which allows a quality demonstration of the characteristics of the regulatory processes.

The numerical solution of the obtained system of equations was performed using the difference scheme based on the fourth-order Runge-Kutta method with a fixed step-length. The identification of model parameters was carried out for the bacterial infection conditions (using streptococcal lung infection as an example) and changes in monocyte production by the bone marrow under the impact of technogenic chemical factors in order to demonstrate quality results allowing an assessment of environmental impact on human health through regulatory system effects.

Thus, the presented model adequately describes the processes of bacterial infection development taking into account the impact of environmental chemical factors. Although the studied structure of interaction between the neuroendocrine and immune system elements is far from being complete and contains only part of the regulatory mechanisms, it is possible to talk about the development of a basic model which reflects the essence of the multicomponent interaction between the regulatory systems in inflammatory responses of bacterial origin and which is ready to be complicated by the introduction of additional parameters and relationships. Accordingly, a future work is planned to expand the component content of the model with the inclusion of adaptive immune responses and antiviral defense mechanisms and possibly include analysis of the dependence of the incidence of infectious diseases on chemical contamination at the population level.

## Figures and Tables

**Figure 4:**
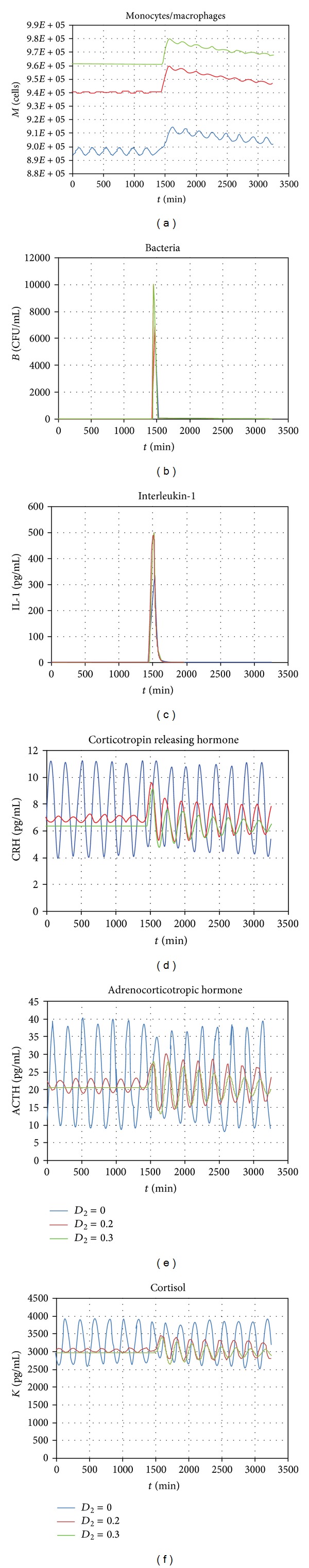
Charts showing changes in (a) macrophage count, (b) bacterial count, (c) IL-1 levels in blood, (d) corticotropin releasing hormone levels in blood, (e) adrenocorticotropic hormone levels in blood, and (f) cortisol levels in blood. The charts show time in hours on the horizontal axis where the curve's starting point corresponds to the point of time of 24 hours before bacterial invasion. The vertical axis shows an indicator's value obtained by a computer simulation. Different colors represent each of the three simulated scenarios with various functional damages to the hypothalamus.

**Figure 5:**
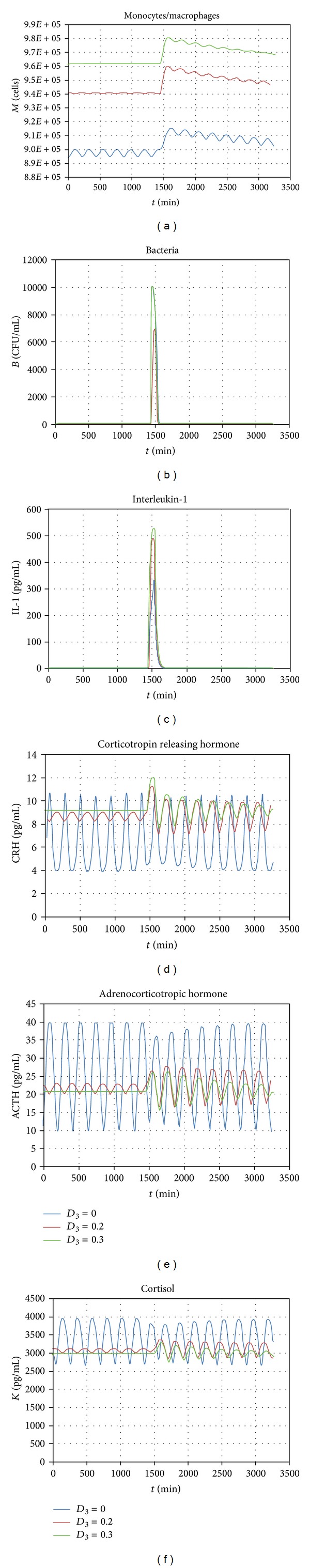
Charts showing changes in (a) macrophage count, (b) bacterial count, (c) IL-1 levels in blood, (d) corticotropin releasing hormone levels in blood, (e) adrenocorticotropic hormone levels in blood, and (f) cortisol levels in blood. The charts show time in hours on the horizontal axis where the curve's starting point corresponds to the point of time of 24 hours before bacterial invasion. The vertical axis shows an indicator's value obtained by a computer simulation. Different colors represent each of the three simulated scenarios with various functional damages to the pituitary gland.

**Figure 6:**
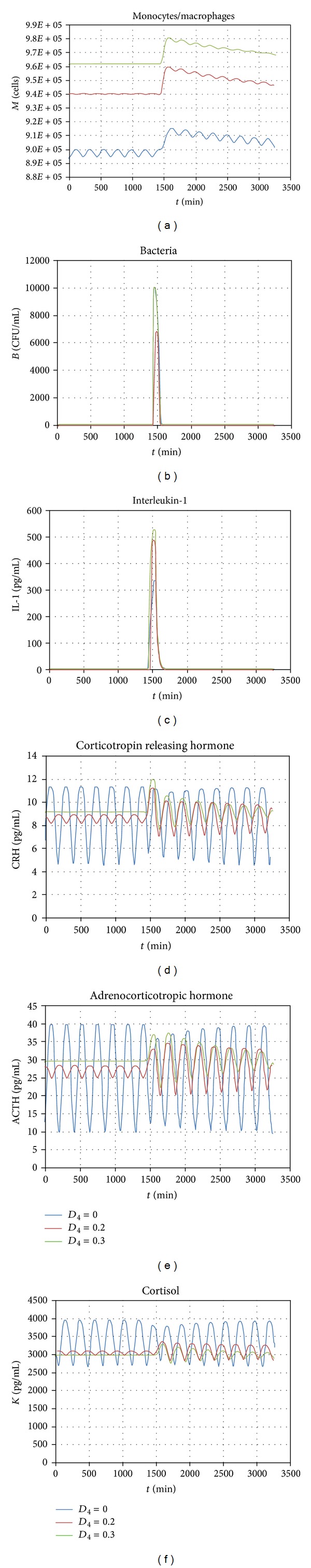
Charts showing changes in (a) macrophage count, (b) bacterial count, (c) IL-1 levels in blood, (d) corticotropin releasing hormone levels in blood, (e) adrenocorticotropic hormone levels in blood, and (f) cortisol levels in blood. The charts show time in hours on the horizontal axis where the curve's starting point corresponds to the point of time of 24 hours before bacterial invasion. The vertical axis shows an indicator's value obtained by a computer simulation. Different colors represent each of the three simulated scenarios with various functional damages to the adrenal glands.

**Table 1:** Model parameters and data sources.

Parameter	Value	Source
*M*_max⁡_	1.3 · 10^6^	Smith et al., 2011 [5]
*β*_1_	480	Coggle and Tarling, 1982 [27]
*β*_2_	2	In-house research
*β*_3_	0.000355	Blusse van Oud Alblas and Van Furth, 1979 [28]
*β*_4_	1	In-house research
*c*	3200	In-house research
*n*	5	In-house research
*τ*	19	In-house research
*P*_max⁡_	2.3 · 10^8^	Smith et al., 2011 [5]
*α*_1_	0	In-house research
*α*_2_	0.0188	Todar, 2002 [29]
*α*_3_	2.62	Hampton et al., 1994 [30]
*α*_4_	0.01	Smith et al., 2011 [5]
*γ*_1_	0.00004	Bergeron et al., 1998 [31]
*γ*_2_	1	In-house research
*γ*_3_	0.04	Gloff and Wills, 1992 [32]
λ_1_	2.6543	Vinther et al., 2011 [33]
λ_2_	1	In-house research
λ_3_	0.001	In-house research
λ_4_	0.17329	Felig and Frohman, 2001 [34]
μ_1_	0.191	Vinther et al., 2011 [33]
μ_2_	1	In-house research
μ_3_	0.034832	Carroll et al., 2007 [35]
ν_1_	1.3	Vinther et al., 2011 [33]
ν_2_	0.0090726	Carroll et al., 2007 [35]
*a*_0_	0.042	In-house research
*b*_0_	0.0000004	In-house research
*p*_0_^*N*^	0.1	In-house research
*M*(*t*_0_)	9 · 10^5^	In-house research
*P*(*t*_0_)	10^4^	In-house research
IL-1(*t*_0_)	0	In-house research
CRH(*t*_0_)	6.9	In-house research
ACTH(*t*_0_)	24.451	In-house research
*K*(*t*_0_)	3805.8	In-house research

## Appendix

### Scenarios with Various Functional Damages to the Hypothalamus, the Pituitary, and the Adrenal Glands

See Figures 4, 5, and 6.
